# Novelty Detection Classifiers in Weed Mapping: *Silybum marianum* Detection on UAV Multispectral Images

**DOI:** 10.3390/s17092007

**Published:** 2017-09-01

**Authors:** Thomas K. Alexandridis, Afroditi Alexandra Tamouridou, Xanthoula Eirini Pantazi, Anastasia L. Lagopodi, Javid Kashefi, Georgios Ovakoglou, Vassilios Polychronos, Dimitrios Moshou

**Affiliations:** 1 Laboratory of Remote Sensing and GIS, Faculty of Agriculture, Aristotle University of Thessaloniki, 54124 Thessaloniki, Greece; tamouridoualex@gmail.com (A.A.T.); georobak@hotmail.com (G.O.); 2 Agricultural Engineering Laboratory, Faculty of Agriculture, Aristotle University of Thessaloniki, 54124 Thessaloniki, Greece; renepantazi@gmail.com (X.E.P.); dmoshou@auth.gr (D.M.); 3 Plant Pathology Laboratory, Faculty of Agriculture, Aristotle University of Thessaloniki, 54124 Thessaloniki, Greece; lagopodi@agro.auth.gr; 4 USDA-ARS-European Biological Control Laboratory, Tsimiski 43, 7th floor, 54623 Thessaloniki, Greece; jkashefi@ars-ebcl.org; 5 Geosense S.A., Filikis Etairias 15-17, Pylaia, 55535 Thessaloniki, Greece; vpoly@geosense.gr

**Keywords:** weeds, UAS, RPAS, one-class, machine learning, remote sensing, geoinformatics

## Abstract

In the present study, the detection and mapping of *Silybum marianum (L.) Gaertn.* weed using novelty detection classifiers is reported. A multispectral camera (green-red-NIR) on board a fixed wing unmanned aerial vehicle (UAV) was employed for obtaining high-resolution images. Four novelty detection classifiers were used to identify *S. marianum* between other vegetation in a field. The classifiers were One Class Support Vector Machine (OC-SVM), One Class Self-Organizing Maps (OC-SOM), Autoencoders and One Class Principal Component Analysis (OC-PCA). As input features to the novelty detection classifiers, the three spectral bands and texture were used. The *S. marianum* identification accuracy using OC-SVM reached an overall accuracy of 96%. The results show the feasibility of effective *S. marianum* mapping by means of novelty detection classifiers acting on multispectral UAV imagery.

## 1. Introduction

Mapping weed patches is an important aspect of effective site-specific application of herbicides. Remote sensing is a source of data that offers full field coverage at various spatial, spectral and temporal resolutions [1]. However, successful automated detection of weeds on remotely sensed images may be hindered by spectral, textural and shape similarities with the crop. Zhang et al. [2] produced a ground-level, hyperspectral weed mapping method for black nightshade and pigweed in tomato fields. Bayesian classifiers were developed for each season that reached high levels of accuracy with cross-validation species pixel classification (92%). Cross-season validation of single season-based classifiers proved that accuracy was affected by changes in the NIR. A machine learning algorithm was produced that utilised three artificial intelligence (AI) season “expert” classifiers in a multi-season, multi-classifier method. This approach achieved a recognition rate of 95.8%, slightly higher than a global calibration approach with multi-season species discrimination accuracies from 90% to 92.7%.

Weed identification methods are based on a variety of image processing techniques, including morphology, spectral features and visual shape. Åstrand and Baerveldt [3] worked on robot vision-based perception for weed identification and control. Their device carried a grey-scale camera with a near-infrared filter for high-contrast images for row detection and a colour camera to identify crop plants. The lateral offset error was ± 1 cm and the error measured with the downward-looking camera at the weeding tool position was ± 0.5 cm. The colour camera efficiently recognised crops via image segmentation and discriminated weeds from crops.

Techniques from the field of machine learning have been effectively applied in land cover classification with remote sensing [4]; meanwhile, during the last years the precision crop protection community has used the advantages of machine learning [5]. Support Vector Machines (SVMs) [6] are among the most prominent machine learning techniques which are utilised for pattern recognition and regression and can be characterised as unique and faster than other computational intelligence techniques due to the fact that they are always capable of converging to a global minimum, and have a simple geometric explanation. SVMs have the ability to effectively handle large volumes of data or attributes. Thus, they can be useful in processing very high-resolution UAV images.

Sluiter [7] studied a wide range of vegetation classification techniques using remote sensing imagery in the Mediterranean region. It was proven that random forests as well as support vector machines demonstrated better performance among traditional classification methods. Regarding feature extraction, Sluiter [7] utilised the spatial domain, more precisely, the spectral information per-pixel and of neighbouring pixels to classify natural vegetation types from remote sensing imagery. The contextual technique SPAtial Reclassification Kernel (SPARK) was implemented, and successfully detected vegetation types, which other per-pixel-based methods failed to detect. Im and Jensen [8] applied a three-channel neighbourhood correlation image model for monitoring vegetation changes using the pixel relations, and their contextual neighbours achieved similar results. More recently Dihkan et al. [9] utilised satellite imagery for mapping tea plantations based on support vector machines. In this study, a three-step approach was proposed and implemented on a test area with high slope, in order to discriminate tea plantations from other types of vegetation. The overall accuracies reached over 90% for land use/land cover mapping.

Novelty detection and machine learning techniques can be collectively recruited to detect abnormal situations. Crupi et al. [10] proposed a novel neural network based technique for the validation of vibration signatures and the indication of a fault occurrence. The model was trained using the data set consisting only of normal examples. Abnormalities, corresponding to fault occurrences, were detected as noteworthy deviations that varied from the normality description. In particular, one class classifiers are characterised by the following behaviours [11]: (i) the only data available correspond to the target class and not to the outlier class; (ii) data coming from the target class are the only means of estimating the separating threshold between the two classes; and (iii) describing a threshold encircling the target class is a challenge.

*Silybum marianum (L.) Gaertn*. is a weed that is hard to eradicate. It is found in cultivated areas and pastures [12]. The leaves can cause symptoms of toxicity to livestock due to the build-up of nitrates [13]. Khan et al. [14] have demonstrated the allelopathic consequences of *S. marianum* on various cultivated species. Herbicides are expensive and they pollute the ecosystems, rendering it necessary to map the spatial distribution of *S. marianum* and define appropriate management strategies.

Previous efforts to map *S. marianum* with UAV images include Tamouridou et al. [15], who identified the optimum resolution for mapping *S. marianum* patches with UAV images, Pantazi et al. [16] who tested three hierarchical self-organising map classifiers, namely, the supervised Kohonen network, counter-propagation artificial neural network, and XY-Fusion network. Efforts to map other weeds using UAV images include Peña et al. [17], who used UAV images acquired at six regions of the visible and near-infrared spectrum and identified weeds from wheat field with Object Based Image Analysis (OBIA) with an overall accuracy of 86%. Higher accuracy rates were achieved when mapping weeds in a sunflower field with RGB images taken by a UAV [18]. Various spatial resolution levels taken from different altitudes using two sensors on board a vertical take-off and landing UAV, were tested in relation to the needs of different weed management applications [19].

The aim of this work is to evaluate the proposed novelty detection classifiers in recognising *S. marianum* weed patches as a target class and detecting all other vegetation as outliers. The algorithms were applied on multispectral images acquired by a camera on board a fixed wing UAV. The features that were employed were constructed by combining spectral and textural information.

## 2. Materials and Methods

### 2.1. Experimental Study Location

**Figure 1:**
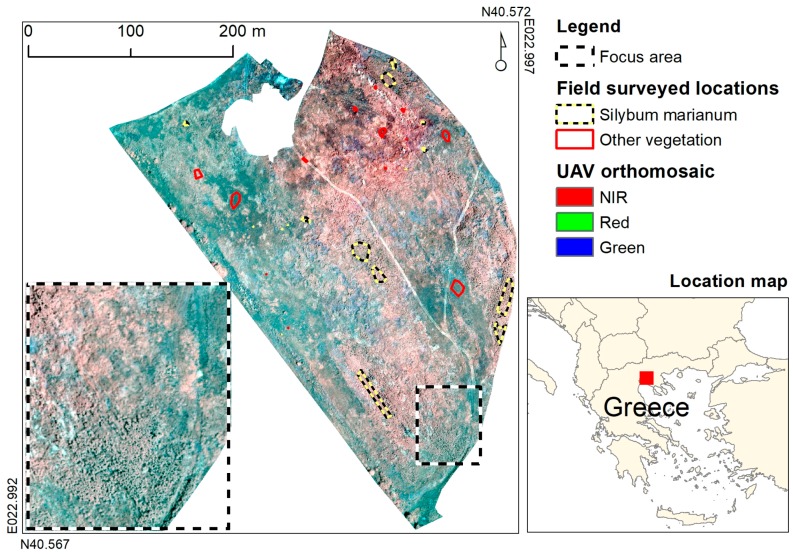
Study area, UAV orthomosaic, focus area, and field surveyed locations.

The experimental study took place at a 10.1 ha field located in Thessaloniki, Greece (Figure 1). The topography of the field is almost level and the elevation is 75 m. The field was cultivated with cereals until 1990, and now graminaceous weeds have colonised it, together with sizeable patches of *S. marianum*. Other dominant weeds include *Avena sterilis* L., *Bromus sterilis* L., *Solanum elaeagnifolium* Cav., *Conium maculatum* L., *Cardaria draba* L. and *Rumex* sp. L.

### 2.2. Datasets

Imagery was acquired on 19.05.2015, a sunny day with a light breeze not more than 3 m∙s^−1^ during the flight. A fixed wing UAV was used (senseFly eBee) with camera payload Canon S110 NIR that captures spectral bands that correspond to green (560 nm, Full-Width Half-Maximum (FWHM): 50 nm), red (625 nm, FWHM: 90 nm) and near-infrared (850 nm, FWHM: 100 nm). The acquisition time frame was between 11:00 am and 12:00 pm to minimise ground shadows and maximise the reflectance effect. The camera was set to shutter priority at 1:2000 s shutter speed. To adapt to potential ground light variations and reflections, we let the camera decide the correct aperture and sensor sensitivity.

Flight was set at a height of 115 m above take-off (relative height), in flight paths that achieved 75% overlap and 70% sidelap. The above setup gave ground sampling distance up to 0.04 m and each image footprint was 160 m × 120 m. The total number of captured images was 55; all of them stored in raw format to avoid radiometric distortion due to file compression. During capture, the drone autopilot was registering coordinates for each image by its GPS.

After landing, the autopilot log file was matched with the acquired images. In order to provide precise geolocation, we measured six targets that were laid down on the ground prior to the flight with a dual frequency Spectra Precision SP80 GNSS receiver. Each target was marked with an absolute accuracy down to 2 cm, using a real time kinematic (RTK) method. Photogrammetric processing was done with Pix4D mapper Pro, photogrammetry software that produced an orthomosaic, a 2.5D digital surface model (DSM) with a typical accuracy of 2 × ground sampling distance (GSD) horizontal and 3 × GSD vertical. The orhomosaic and the DSM were further resampled to a final resolution of 0.5 m that was adequate for mapping the weed patches, while avoiding noise introduced to the classifiers from finer resolutions [15]. In addition to the three spectral bands, the texture of NIR band was created using the local variance algorithm with a moving window of 7 × 7 pixels, and added to the orthomosaic as inputs to the classification algorithms.

The location of patches of *S. marianum* and of other prevailing vegetation types were recorded on a Trimble GeoXH GPS (accuracy ~0.3 m), in order to help to build the training sets that were needed for image classification. As *S. marianum* patches were impenetrable, their outline was marked on the GPS. To ensure equal class representation, a calibration dataset was created consisting of two sets of 1434 pixels each for classes “*S. marianum*” and “other vegetation” (2868 pixels in total). From this calibration dataset, a random subset of 70% (2008 pixels for both classes) was used for algorithm training and the rest 30% (860 pixels for both classes) for testing the result. This dataset was common to all classification algorithms.

**Figure 2:**
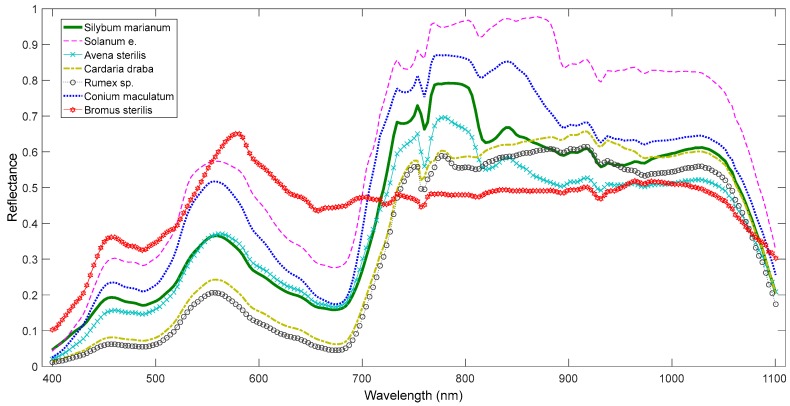
Spectra corresponding to each vegetation type.

For the evaluation of weed recognition, spectral signatures were acquired from *S. marianum* plants, and also from randomly chosen plants of other species (Figure 2) on 29 May 2015 using a UniSpec-DC spectrometer (PP Systems, Inc., Amesbury, MA, USA). It is a handheld dual channel spectrometer, simultaneously measuring incident and reflected light. The spectrometer’s wavelength range is 310–1100 nm (visible and near-infrared) and its spectral resolution is less than 10 nm.

It is illustrated in Figure 2 that various plant species demonstrate similar spectral behaviour, rendering the discrimination a challenge. For instance, *S. marianum* and *A. sterilis* presented very strong similarities in the visible range of the spectrum, in contrast to the NIR area.

### 2.3. Novelty Detector Classifiers

#### 2.3.1. OC-SVM

To achieve One Class SVM Classification, a suitable description of SVM as a model to describe only target data was introduced by Tax and Duin [20] in the form of Support Vector Data Description (SVDD). Given a data set of targets $\left\{x_{i}\right\},i=1,2,3,.....,N$ used for training, the goal of SVDD is to implement by learning a decision function to decide whether an example is either a target or an outlier. SVDD is based on the hypothesis that target data are encircled by a close boundary in the feature space. The simplest type of closed boundary is a supersphere, which is simple to describe with only two parameters: the centre a and the radius *R*. A supersphere is asked to cover the given target data set members but with the smallest radius *R*. This optimization problem is constructed as follows:

(1) $$\begin{array}{l} \min R^{2}+C\sum_{i=1}^{N}\xi_{i}\\ s.t.||x_{i}-a{\left|\right|}^{2}\leq R^{2}+\xi_{i},\;i=1,2,.....,N \end{array}$$

where *C* assigns a penalty to the loss term and *ξ_i_* are the slack variables, the value of *C* is determined by the expected upper function bound *ν* on misclassified targets:

(2) $$C=\frac{1}{N\cdot v}$$

*α* is computed:

(3) $$\alpha =\sum_{i=1}^{N}\alpha_{i}x_{i},\;0\leq \alpha_{i}\leq C$$

The value of *α_i_* can be delivered into three categories:

$$\alpha_{i}=0\Rightarrow ||x_{i}-\alpha ||<R^{2},$$

$$0<\alpha_{i}<C\Rightarrow ||x_{i}-\alpha |{|}^{2}=R^{2},$$

$$\alpha_{i}=C\Rightarrow ||x_{i}-\alpha |{|}^{2}<R^{2}.$$

To predict an example *ν*, the distance between ν and α is computed:

(4) $$||\nu -\alpha |{|}^{2}\leq R^{2}\Rightarrow \nu \;\mathrm{is}\;\mathrm{target}\;||\nu -\alpha |{|}^{2}>R^{2}\Rightarrow \nu \;\mathrm{is}\;\mathrm{outlier}$$

**Figure 3:**
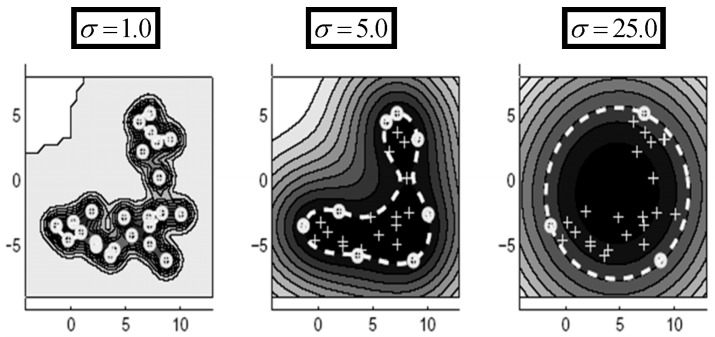
The influence of the RBF spreading parameter on the behaviour of the one class SVM [20].

The OC-SVM develops a model by being trained in using normal data conforming to the SVDD description. At the second stage, it allocates test data based on the ocurring deviation from normal calibration data as being either normal or outlier [21]. The effect of the Radial Basis Function's (RBF) spreading parameter in $K(x,z)=\exp\{-{\left\|x-z\right\|}^{2}/\sigma^{2}\}$ can be determined by considering that a sizeable spread indicates a linear class of target data while on the other hand, many support vectors joint with a small spread indicate a highly nonlinear case as is illustarted in Figure 3. A spread parameter equivalent to 2.5 yielded the best results in the *S. marianum* detection presented here. The threshold for accepting outliers was set at 5%.

#### 2.3.2. One Class Self-Organizing Map (OC-SOM)

An OC-SOM is trained using normal operation data. Consequently, the feature vector that matches to a new measurement is inspected in order to evaluate its similarity to the weight vectors of each other map unit. If the smallest distance surpasses a predetermined threshold, it is expected that the process denotes a fault situation. This result stems from the assumption that quantisation faults exceeding a certain value are associated with operation points external to the training data region. Depending on the magnitude of deviation from the normal state, a degradation index can be designed. The OC-SOM [11] creates a model from healthy data and successively classifies new data conferring to its deviation from the healthy baseline condition. During novelty recognition, novel instances from feature combinations (in the current case the features from the multispectral image) of not definable state are used to form the input to the network, while the SOM algorithm chooses the best matching unit (BMU). In Saunders and Gero [22], if the quantisation error that is the outcome from the appraisal between the new exemplar data (x*^NEW^*) and BMU is larger than a pre-specified threshold (*d*) then the example is considered as novel. Equation (5) represents the minimum distance for the BMU and inspects it against the threshold.

(5) $$\min(\sum_{j=0}^{n-1}{\left(x_{j}^{NEW}-m_{i}\right)}^{2})>d,\;i\in M$$

where *M* denotes the SOM grid of neurons.

There are several heuristics to define a threshold based on the effectiveness of the threshold and the precise structure of the data set. A way to define a threshold (*d*) depends on the resemblance between the SOM centroid vectors and the baseline indicating training vectors selected as BMUs which determines the quantisation error. These distances are calculated from Equation (6):

(6) $$\mathrm{distances}=\min(\sum_{k=0}^{N-1}{(x_{k}^{TARGET}-m_{i})}^{2}),\;i\in M$$

**Figure 4:**
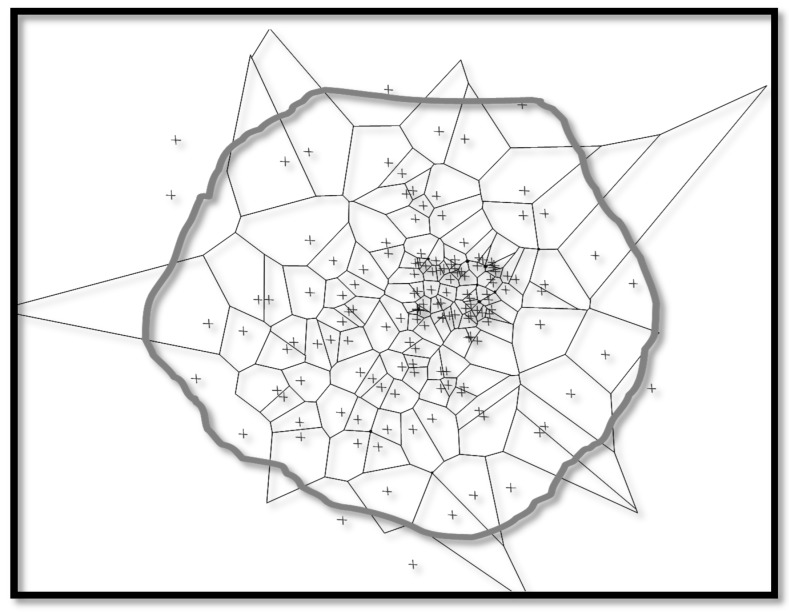
Target dataset regions and Voronoi polygons and the threshold perimeter for OCSOM. Target data defined by the threshold are resident inside the grey border line.

The threshold is programmed in pseudo-code which is presented below:

Data_distances_sorted = sort(distances);
Fraction = round(fraction_targets × length(target_set));
Threshold = (Data_distances_sorted(fraction) + Data _distances_sorted(fraction + 1))/2;

By the threshold’s selection designed to represent a fraction of distances from the whole training set, an assumption is to get distance values in order to represent the codebooks data vectors more faithfully, especially when the distances are formed. Hence, the fraction error represents a subset of distances that can isolate outlier values following a specific distribution. For instance, if the 99% fraction of the distances that separate data and codebooks is chosen to belong to the dataset, the determination of a descriptive hypersphere is direct with a radius that covers 99% of the data. The remaining 1% matches to outliers since they are located exterior to the target set description area. In Figure 4, it is shown that different areas are defined by the threshold relative to the BMUs. The BMUs define Voronoi polygons representing OCSOM neuron domains. It is clear that points selected as residing inside a domain of neuron are situated externally to the threshold-defined polygon which demarcates the border between target and novel data (for illustration purposes, actual data are of high dimension so direct visualisation is not possible). In this paper, an 8 × 8 rectangular OC-SOM gave the best results while 3 × 3 to 20 × 20 were tested. The threshold for accepting outliers was set at 5%.

#### 2.3.3. Auto-Encoders

Auto-encoders are neural networks able to learn a data representation [23,24,25]. They are trained to reproduce input patterns in a mirroring sense as an output (they perform an identity operation). In the auto-encoder architecture, the structure uses only one hidden layer with a number of hidden units which are related to input features. Sigmoidal transfer functions are used.

The auto-encoder networks will successfully reconstruct target objects but with a smaller error compared to outlier objects. With one hidden layer, a (linear) principal component type of solution is the outcome [26]. The auto-encoder network will tend to find a data description similar to the PCA description. To obtain a small reconstruction error from the target set, the network is forced to construct a compact mapping starting from the data into the subspace and structurally coded inside these hidden neurons. The total number of hidden units provides the dimensionality of this subspace. The non-linear transfer functions of the neurons in other layers deem this subspace non-linear.

Auto-encoder methods are flexible, but they inherit several problems from multilayer perceptrons because they are very similar to them [11]. These problems concern sensitivity to improper training parameter selection by the user and predefinition of the Auto-encoder architecture. In this paper, the inputs were the three spectral features and the texture, while 8 hidden units provided the best result ranging from a number of trials in which 3 to 20 were utilised.

#### 2.3.4. OC-PCA

Principal Component Analysis (PCA) (or the Karhunen-Loeve transform) [27] is used for data belonging to a linear subspace. The PCA mapping discovers the orthonormal subspace which represents the variance of the data (in the squared error sense). The optimisation procedure uses eigenvalue decomposition to find the eigenvectors of the covariance matrix of target data. The eigenvectors that are associated to the largest eigenvalues are the principal axis of the d-dimensional data while their direction corresponds to the largest variance. These vectors form an orthonormal basis vectors for the data. The number of basis vectors *M* is adjusted to a user-defined fraction of the variance in the data. The basis vectors W become a d×M matrix. Since they form an orthonormal basis, the total free parameters in the PCA are determined by:

(7) $$n_{freePCA}=\left(\begin{array}{c} d-1\\ M \end{array}\right)$$

When computing the PCA, a zero mean dataset is often assumed. If the data has to be estimated, this operation will add d more free parameters to *n_free__PCA_*.

The reconstruction error of an object *z* [11] is based on the squared distance from the original object and the corresponding mapped version:

(8) $$d_{PCA}(z)={\left\|z-(W{\left(W^{T}W\right)}^{-1}W^{T})z\right\|}^{2}={\left\|z-(WW^{T})z\right\|}^{2}$$

where the second step is feasible since the basis W is orthonormal.

The PCA achieves good accuracy when a clear linear subspace is the case. For very low sample sizes, the data is located in a subspace. If the intrinsic data dimensionality is smaller than the feature size, the PCA can generalize from the low sample size data. If the available data has variance in all feature directions, then in some cases it would be impossible to reduce the dimensionality without reducing the explained variance too much. When data are in separate subspaces, the PCA yields an average subspace representing data in each subspace very poorly. Scaling alters the large variance directions and thus the PCA basis. When data directions are amplified, it will improve the PCA description. On the other hand, when the noise is amplified, it will damage the characterisation. PCA only focuses on the variance of the target set, a drawback is that PCA is not able to include negative examples in the training set. In this paper, a threshold of 10% was set for accepting outliers which gave the best performance.

## 3. Results and Discussion

The performance of the novelty detector classifiers into identifying *S. marianum* and other vegetation types are presented in Table 1. Accuracy assessment was achieved by computing the confusion tables on the validation dataset (860 pixels), with 441 belonging to *S. marianum* and 419 to other vegetation types [28]. All classifiers were characterised by very high overall accuracy, exceeding 90%. OC-SVM performed better, reaching 96% overall accuracy, and equally high user’s and producer's accuracy. OC-SOM had marginally lower overall performance (94.65%) which is explained by the underestimation of 8.39% of *S. marianum* actual observations (omission errors). Auto-encoder had also slightly lower overall accuracy (94.3%), which is attributed to 5.7% of pixels confused between the two categories, leading to a uniformly lower user’s and producer’s accuracy (94%). OC-PCA had lower overall accuracy (90%) which is explained by the underestimation of 11.56% of *S. marianum* actual observations (omission errors), and the overestimation of 8.35% of other vegetation image pixels (commission errors).

**Figure 5:**
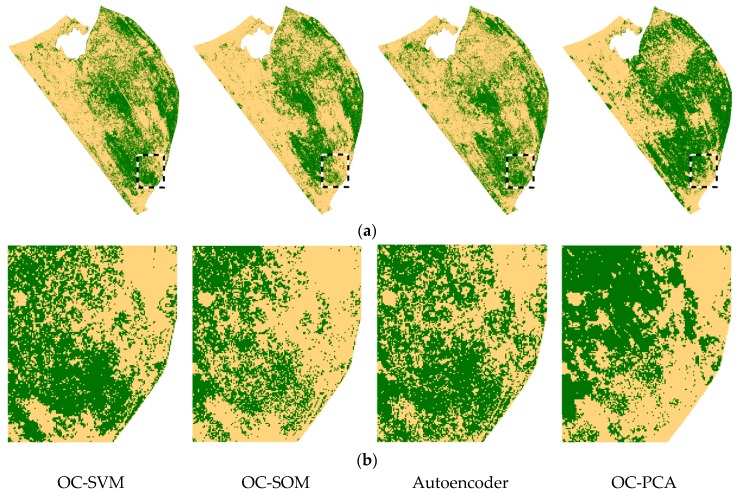
*Silybum marianum* coverage based on four novelty classifiers prediction in the study area (**a**); and in the focus area (**b**) *S. marianum* is green colour and other vegetation is yellow.

The trained OC-SVM, OC-SOM, Autoencoder and OC-PCA models were provided with feature vectors originating from the whole UAV mosaic with 782,838 samples. Each of the input vectors produced a decision corresponding to 1 for *S. marianum* and 2 denoting other vegetation (Figure 5). The weeds map derived from OC-SVM classifier shows large patches of *S. marianum* along the eastern border of the study area, and a high concentration of patches in the central section of the study area, while only few patches are identified in the remaining western part. In the focus area, a large contiguous patch is evident in the southern part, and several densely vegetated patches towards the northwest corner. A slight underestimation of *S. marianum* is evident in the OC-SOM map as compared to OC-SVM, this is most notable in the central north and southeast parts of the study area. This is evident in the focus area, where several gaps have appeared in the large contiguous patch that was mapped with the OC-SVM classifier. In the Autoencoder map, patches of *S. marianum* are missing in the central north part of the study area, while other areas have been incorrectly classified as *S. marianum*. Similar patterns of misclassification appear in the OC-PCA map, where the omission of *S. marianum* patches is more prominent. In the focus area, the large contiguous patch at the southern side is almost entirely missing, while patches at the northwest corner appear denser than those mapped with the other three classifiers.

The vegetation surrounding the *S. marianum* patches consists of graminaceous weeds, forming an even surface with a very smooth texture, in contrast to the *S. marianum* patches, which consist of large individuals with high uneven texture. Thus, texture was a feature contributing to the novelty detection of all classifiers.

The autoencoder and OC-PCA classifiers displayed a notable over-estimation of the *S. marianum* category in the northern part of the study area, which was in fact dominated by *A. sterilis*. This over-estimation can be observed in Figure 5a, and may be due to the fact that *A. sterilis* plants remained in a healthy condition despite the season (late spring), when the rest of the area’s vegetation was in senescence. As a result, the spectral signatures of the two species are highly similar (Figure 2) resulting in the aforementioned confusion. The autoencoder and OC-PCA were especially susceptible to this confusion, as they simply rotate data to new multidimensional axes, while OC-SVM and OC-SOM use support vectors and local neurons, respectively.

All of the examined novelty detection classifiers demonstrated a very high level of performance, with overall accuracies ranging from 90% to 96.05%. Compared to other classifiers used with the same dataset, the parametric maximum likelihood classifier achieved lower overall accuracy of 87% [15], while all hierarchical self-organising map classifiers achieved higher accuracies up to 98.87% [16].

Aiming to employ site specific weed management, classification accuracy is of vital importance since the output maps will consequently be utilised as treatment maps. Furthermore, those thematic output maps can be utilised to monitor the spatial distribution of weeds (in this case *S. marianum*), as well as its variation in time, in the case of weed eradication applications.

Coverage maps on a national and regional level can be created by means of relatively low-cost technology like satellite remote sensing [29]. The aim of utilising satellite imagery is to map out the spatial distribution of larger-scale infestations.

On the other hand, when the aim is to apply treatment practices, high accuracy in detection and classification is imperative, even in cases of low-density infestations. Accordingly, multispectral or hyperspectral imagery is the necessary approach, and the suggested method of acquisition is by employing UAV technology. The acquired data and results can be utilised to detect areas susceptible to weed growth due to favourable conditions, to identify problematic areas where the weeds could spread rapidly, and lastly, to design new and evaluate existing management practices.

## 4. Conclusions

In the current study, it was proven that it is possible to locate *S. marianum* patches with the aid of four novelty detection classifiers. The classifiers were OC-SVM, OC-SOM, OC-PCA and Autoencoder. For the image acquisition, a multispectral camera (green-red-NIR) mounted on a UAV was utilised. As input features for the classifiers, three spectral bands from the camera and the texture, calculated as local variance of the NIR band, were applied. The highest rates of overall accuracy were achieved using OC-SVM network (96.05%), while slightly lower accuracies were scored by OC-SOM (94.65%), OC-PCA (90%) and Autoencoder (94.30%). The results advocate that using one class novelty detectors can be applied operationally for mapping *S. marianum* with UAV for several operations encompassing weed eradication programs.

## Figures and Tables

**Table 1:** Contingency table and optimal parameters of each classifier tested for the identification of *S. marianum* against other vegetation.

Network Prediction					
Classifier (Overall Accuracy %)	Actual Observations	*S. marianum* (Pixels)	Other Vegetation (Pixels)	User’s Accuracy (%)	Producer’s Accuracy (%)
OC-SVM σ = 2.5 (96.05)	*S. marianum*	416	25	97.88	94.33
	Other vegetation	9	410	94.25	97.85
OC-SOM, 8 × 8 (94.65)	*S. marianum*	404	37	97.82	91.61
	Other vegetation	9	410	91.72	97.85
Autoencoder, 8 hidden (94.30)	*S. marianum*	416	25	94.55	94.33
	Other vegetation	24	395	94.05	94.27
OC-PCA (90.00)	*S. marianum*	390	51	91.76	88.44
	Other vegetation	35	384	88.28	91.65
